# Regulatory Mechanisms Controlling Maturation of Serotonin Neuron Identity and Function

**DOI:** 10.3389/fncel.2017.00215

**Published:** 2017-07-19

**Authors:** William C. Spencer, Evan S. Deneris

**Affiliations:** Department of Neurosciences, Case Western Reserve University Cleveland, OH, United States

**Keywords:** 5-HT neuron, development, transcription factors, terminal selector, neurotransmitter, RNA-seq

## Abstract

The brain serotonin (5-hydroxytryptamine; 5-HT) system has been extensively studied for its role in normal physiology and behavior, as well as, neuropsychiatric disorders. The broad influence of 5-HT on brain function, is in part due to the vast connectivity pattern of 5-HT-producing neurons throughout the CNS. 5-HT neurons are born and terminally specified midway through embryogenesis, then enter a protracted period of maturation, where they functionally integrate into CNS circuitry and then are maintained throughout life. The transcriptional regulatory networks controlling progenitor cell generation and terminal specification of 5-HT neurons are relatively well-understood, yet the factors controlling 5-HT neuron maturation are only recently coming to light. In this review, we first provide an update on the regulatory network controlling 5-HT neuron development, then delve deeper into the properties and regulatory strategies governing 5-HT neuron maturation. In particular, we discuss the role of the 5-HT neuron terminal selector transcription factor (TF) Pet-1 as a key regulator of 5-HT neuron maturation. Pet-1 was originally shown to positively regulate genes needed for 5-HT synthesis, reuptake and vesicular transport, hence 5-HT neuron-type transmitter identity. It has now been shown to regulate, both positively and negatively, many other categories of genes in 5-HT neurons including ion channels, GPCRs, transporters, neuropeptides, and other transcription factors. Its function as a terminal selector results in the maturation of 5-HT neuron excitability, firing characteristics, and synaptic modulation by several neurotransmitters. Furthermore, there is a temporal requirement for Pet-1 in the control of postmitotic gene expression trajectories thus indicating a direct role in 5-HT neuron maturation. Proper regulation of the maturation of cellular identity is critical for normal neuronal functioning and perturbations in the gene regulatory networks controlling these processes may result in long-lasting changes in brain function in adulthood. Further study of 5-HT neuron gene regulatory networks is likely to provide additional insight into how neurons acquire their mature identities and how terminal selector-type TFs function in postmitotic vertebrate neurons.

## 1. Introduction

**Figure 1:**
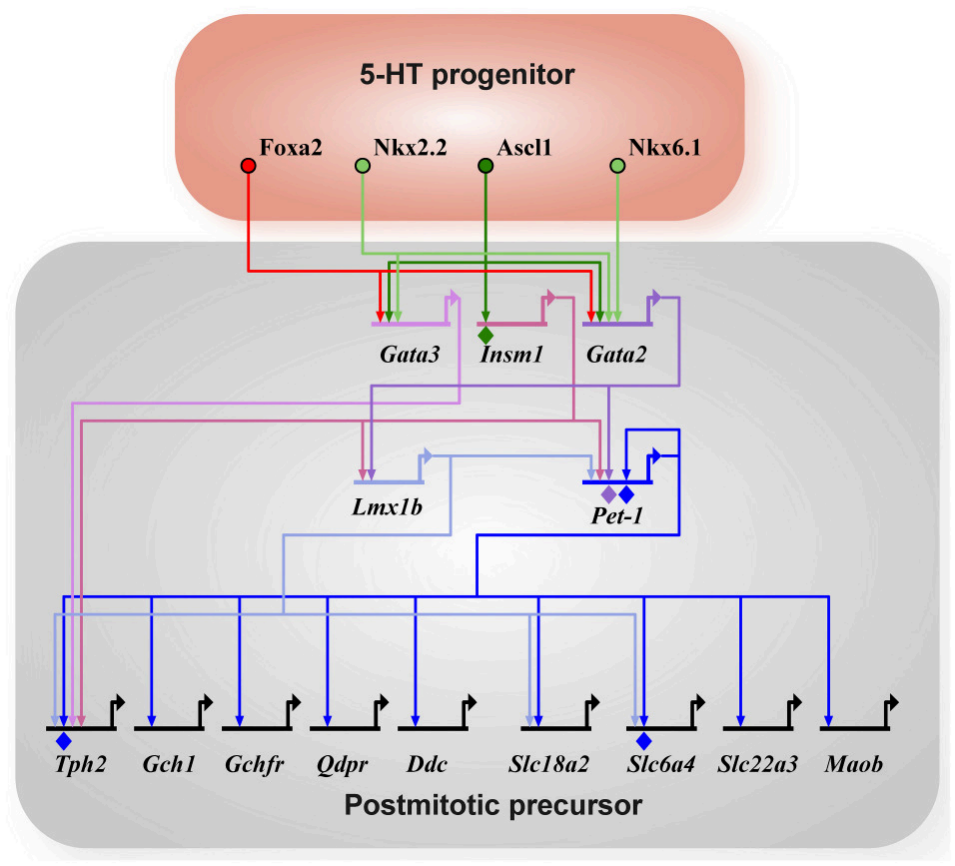
5-HT neuron gene regulatory network. All arrows indicate activation and diamonds indicate direct binding of the transcription factor to the promoter of the target gene as determined by ChIP experiments. 5-HT neuron generation in rhombomere 1 is driven by Foxa2 and Nkx6.1 (in chick). Ascl1 acts as a pro-neural factor activating expression of Gata2, Gata3, and Insm1 in parallel to Foxa2 and Nkx6.1. Terminal differentiation is initiated by Gata3, Lmx1b, and Pet-1 through the activation of the 5-HT neuron gene battery (*Tph2, Gch1, Gchfr, Qdpr, Ddc, Slc18a2, Slc6a4, Slc22a3, Maob*). *Tph2*, tryptophan hydroxylase 2; *Gch1*, GTP cyclohydrolase 1; *Gchfr*, GTP cyclohydrolase 1 feedback regulator; *Qdpr*, quinoid dihydropteridine reductase; *Ddc*, AADC, aromatic L-amino acid decarboxylase; *Slc18a2*, Vmat2, vesicular monoamine transporter 2; *Slc6a4*, SERT, 5-HTT, serotonin transporter; *Slc22a3*, Oct3, organic cation transporter 3; *Maob*, monoamine oxidase B.

Serotonin (5-hydroxytryptamine; 5-HT) is a monoamine neurotransmitter produced by various clusters of neurons in the raphe of the vertebrate midbrain and hindbrain (B1-B9, Dahlström and Fuxe, 1964). 5-HT has been broadly associated with neuropsychiatric disorders such as major depressive disorder (Aghajanian and Marek, 2000; Hensler, 2006). 5-HT neurons are defined by the ability to synthesize, package, release, autodetect, and inactivate 5-HT. The 5-HT biosynthetic pathway involves hydroxylation of tryptophan to 5-hydroxytryptophan (5-HTP) by the tryptophan hydroxylase enzyme Tph2, and a subsequent decarboxylation to 5-HT by the L-aromatic amino acid decarboxylase enzyme Aadc (*Ddc*) (Rahman and Nagatsu, 1982; McKinney et al., 2005). 5-HT is packaged into vesicles by the vesicular monoamine transporter Vmat2 (Fon et al., 1997). After neurons fire and release 5-HT into the synaptic cleft, the serotonin reuptake transporter (Sert, 5-HTT) transports 5-HT back into the neuronal cytoplasm or 5-HT is degraded by monoamine oxidase (Maoa or Maob) thus inactivating the 5-HT signal (Levitt et al., 1982; Blakely et al., 1991; Tipton et al., 2004). Activation of this battery of genes is necessary for acquisition of 5-HT transmitter identity (Figure 1). The number of 5-HT neurons is minuscule in relation to the total number of neurons in the brain, with approximately 26,000 in the mouse and 400,000 in humans (Ishimura et al., 1988; Baker et al., 1990; Hornung, 2003). Despite the small number of cells, the connectivity pattern of 5-HT neurons is vast with axons projecting throughout the entire central nervous system (CNS, Lidov and Molliver, 1982). Given how intertwined 5-HT neurons are within the CNS, it is unsurprising that 5-HT neurotransmission has many functions that if perturbed could result in mental health issues later in life (Gaspar et al., 2003; Lesch et al., 2012).

**Figure 2:**
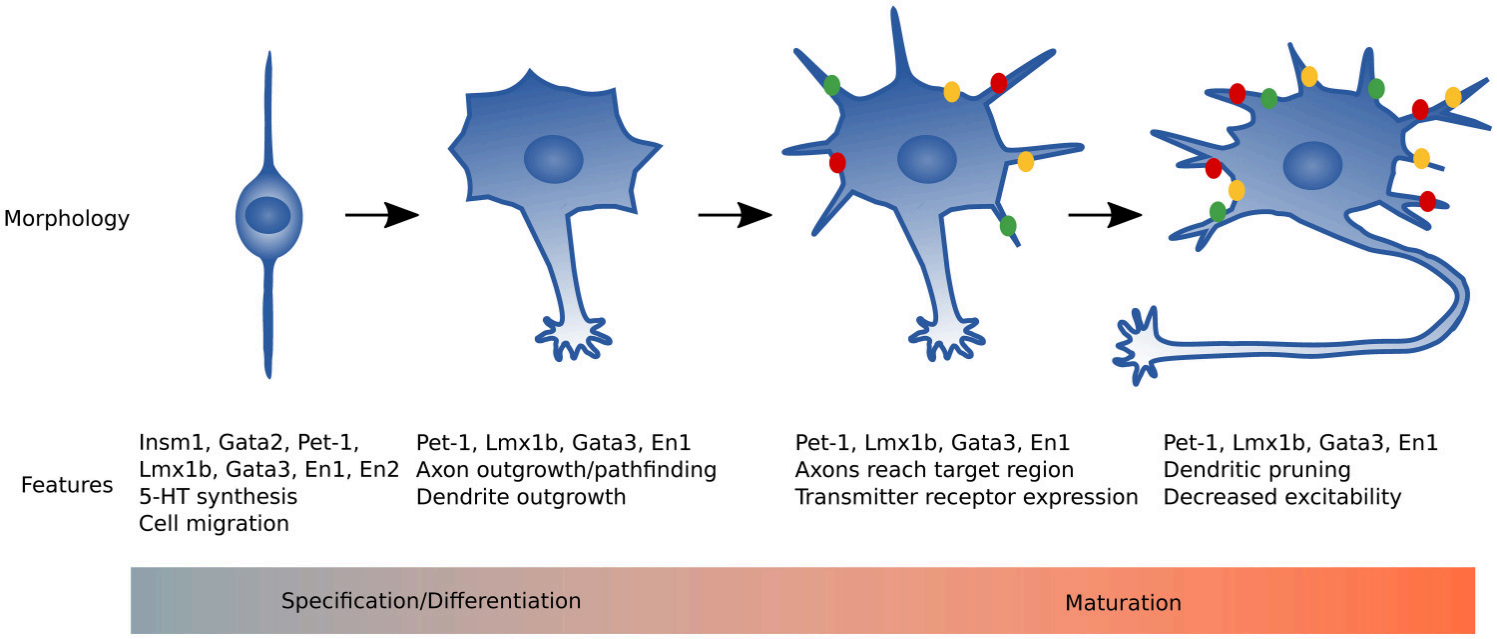
Summary of 5-HT neuron maturation. Immature 5-HT neurons have a simple bipolar morphology and utilize nuclear translocation to migrate to the proper raphe location. Pet-1, Lmx1b, and Gata3 activate the 5-HT gene battery to begin 5-HT synthesis. Axons of immature 5-HT neurons extend, growing rostrally and caudally, and reach target regions around birth in rodents. In the early postnatal period dendrites are relatively short with moderate branching and most genes required for mature neuronal function are expressed. From early postnatal through 3 weeks of life, dorsal raphe neuron dendrites lengthen then retract and reduce branch number by 2 months of age. Green, yellow, and red dots represent transmitter receptors that increase expression levels during maturation (e.g., Htr1a, Adra1b, and Lpar1).

Essentially all 5-HT neurons are born and start to synthesize 5-HT by E11 in the mouse (Hendricks et al., 2003; Pattyn et al., 2003). These immature neurons express the core features of 5-HT neurons represented in the the 5-HT gene battery (Figure 1), yet they still must elaborate axonal and dendritic processes, establish synaptic connectivity with afferents and postsynaptic targets, and acquire adult firing characteristics. Thus, the time between E12 and at least 3 weeks of life represents a maturation stage where 5-HT neurons develop the complete repertoire of adult characteristics (Figure 2). In this review, we will discuss recent advances in our understanding of the regulatory strategies controlling 5-HT neuron maturation.

## 2. Summary of the regulatory network controlling 5-HT neuron development

The gene regulatory network involved in the specification and differentiation of 5-HT neurons is well-described elsewhere (Kiyasova and Gaspar, 2011; Deneris and Wyler, 2012), thus a brief summary will be provided (Figure 1). The homeodomain TF, Nkx2.2, and the forkhead box TF, Foxa2, are the two main transcription factors induced by Sonic hedgehog (Shh) signaling in the ventral rhombencephalon. 5-HT neurons derived in rhombomere 1 do not depend on Nkx2.2 and are induced by the homeodomain TF Nkx6.1 (only in chick) and Foxa2, whereas generation of most other 5-HT neurons depends on Nkx2.2 with the exception of a subset in B1, B2, and B3 (Briscoe et al., 1999; Jensen et al., 2008). Foxa2 is required to repress visceral motor neuron fate in the rostral rhombomeres. The Nkx TFs activate expression of the GATA TFs, Gata2 and Gata3, in postmitotic precursors. The proneural bHLH TF, Ascl1, also induces Gata2 and Gata3 directly, as well as, the ETS TF Pet-1 and the Lim-homeodomain TF Lmx1b through the Zinc-finger TF, Insm-1. In parallel, Gata2 directly binds the promoter of Pet-1 and activates Pet-1 expression (Krueger and Deneris, 2008). Thus, Pet-1, Lmx1b, and Gata3 are activated in postmitotic precursors to initiate terminal differentiation through activation of the 5-HT neuron gene battery. Pet-1 and most likely Lmx1b and Gata3 are terminal selector-type TFs as they are continuously expressed and function to activate the core 5-HT neuron identity. In addition to activation of the 5-HT neuron gene battery, Pet-1 also shapes 5-HT neuron identity through transcriptional repression (see section 4.3 below). Each of these TFs do not require the others for their initial activation and all three maintain expression throughout life. In a recent study, it has now been shown that Gata2 and Gata3 have an additional role in generating a subtype of 5-HT neurons in the DRN that express the vesicular glutamate transporter (*Slc17a8*, vGlut3) (Haugas et al., 2016).

A potentially important translational application of the 5-HT neuron gene regulatory network is the ability to directly drive differentiation of stem cells to 5-HT+ neurons, or even further, to trans-differentiate another cell-type (e.g., fibroblasts) into 5-HT+ neurons. Several recent studies have tested exogenous factors and multiple combinations of transcription factors known to have a role in driving 5-HT neuron specification (Dolmazon et al., 2011; Vadodaria et al., 2016; Xu et al., 2016). An interesting first test showed the capability of Lmx1b to induce mouse embryonic stem cells to differentiate into 5-HT neurons (Dolmazon et al., 2011). In the presence of Shh, an inducible Lmx1b construct was able to activate expression of Tph2, Sert, and Pet-1, however 5-HT levels were not determined under those conditions. Overexpression of Lmx1b was able to bypass the need for treatment with FGF4 and FGF8 to induce Nkx2.2 and Nkx6.1, which activate Gata2 and Gata3. Although overexpression of Lmx1b was able to induce a 5-HT neuron-like identity, additional experiments would be required to fully determine the complete 5-HT cell fate using this approach. Two additional studies applied a similar approach by expressing multiple TFs and were able to trans-differentiate human fibroblasts into 5-HT+ neurons (induced 5-HT neurons, iSN; Vadodaria et al., 2016; Xu et al., 2016). Vadodaria et al. (2016) first overexpressed the neurogenic factors *ASCL1* and *NGN2* in human fibroblasts to induce neuronal differentiation, then induced expression of *NKX2.2, FEV* (human ortholog of Pet-1), *GATA2*, and *LMX1B* resulting in 61% of iSNs staining for TPH vs. 8% for control induced neurons after 3 weeks in culture. However, a low percentage were immunopositive for 5-HT (the fraction of 5-HT+ cells was not disclosed). An additional 3 weeks in culture resulted in 38% 5-HT+ neurons. RNA sequencing analysis of iSNs confirmed expression of many serotonergic genes at 3 weeks in culture. Xu et al. (2016) used a doxycycline-inducible expression system to overexpress *ASCL1, FOXA2, FEV*, and *LMX1B* in human primary embryonic fibroblasts. By inducing expression of the four factors and knocking down p53 with an shRNA for 7 days, then removing dox from the culture media, ~25% of total cells were 5-HT+ at day 12. Immunostaining and qRT-PCR indicated several serotonergic genes and general neuronal genes were expressed at days 6 and 25 of culture. Both Vadodaria et al. (2016) and Xu et al. (2016) showed the iSNs were functional neurons and released 5-HT into the culture media. These studies show that utilizing knowledge of the 5-HT neuron gene regulatory network to trans-differentiate fibroblasts is a promising avenue for deriving human patient-specific 5-HT neurons that could be used for research into 5-HT relevant neuropsychiatric diseases. However, it is not clear if the induced 5-HT neurons represent fully mature 5-HT neurons. Xu et al. (2016) showed their iSNs responded to 5-HT treatment with increased firing rates, although most mature 5-HT neurons downregulate their firing rate in response to 5-HT or 5-HT_1_a receptor (5-HT1AR) agonists (see Section 4.2.1). The lack of appropriate 5-HT1AR autoregulation could be due to culture conditions or incomplete G-protein signaling pathway downstream of 5-HT1AR. Further efforts into understanding not only the developmental gene regulatory network required to specify 5-HT neurons, but also the regulatory mechanisms driving 5-HT neurons to their complete mature state may help produce induced 5-HT neurons with fully mature functional properties.

## 3. Maturation of Pet-1+/5-HT neurons

### 3.1. Electrophysiological maturation

A major question for understanding neuronal maturation is: when do 5-HT neurons acquire adult physiological characteristics? There have been few studies to date to investigate the functional maturation of Pet-1+/5-HT neurons in the dorsal and median raphe (Rood et al., 2014; Morton et al., 2015). Rood et al. (2014) compared electrophysiological properties of 5-HT neurons at postnatal days 4, 12, 21, and 60 in mice (Table 1). First, the authors compared membrane characteristics within the dorsal and median raphe. They found that dorsal raphe neurons had a more depolarized resting membrane potential at P4 compared to P12 through P60. Membrane resistance was significantly higher at P4, then decreased at P12 and remained steady through P60. Morton et al. (2015) measured electrophysiological properties of putative 5-HT neurons located in the ventromedial dorsal raphe at P5 to P7 and P15 to P17. Morton et al. (2015) did not find the same changes in membrane properties as Rood et al. (2014), but the changes in action potential properties of threshold, peak amplitude, duration, and afterhyperpolarization amplitude were similar. The differences could be due to the mouse strain background, earlier time-point in Rood et al. (2014), or use of a direct or indirect approach of identifying 5-HT neurons (ePet::EYFP in Rood et al., 2014 and vGat::Venus- in Morton et al., 2015).

**Table 1:** Electrophysiological properties of maturing 5-HT neurons (Rood et al., 2014).

**Property**	**Early postnatal**	**Adult**
Resting membrane potential	⇑	⇓
Membrane resistance	⇑	⇓
Firing rate	⇑	⇓
5-HT1a autoreceptor response	−	+
G-protein signaling	⇓	⇑
Action potential threshold	⇑	⇓
Action potential duration	⇑	⇓
Afterhyperpolarization amplitude	⇑	⇓
Afterhyperpolarization duration (lwDRN)	⇓	⇑

*Arrows indicate changes in properties relative to the alternate age. Minus symbol indicates absence and plus symbol indicates presence of property*.

A hallmark feature of 5-HT neurons is autoinhibition of firing through 5-HT1AR. When Rood et al. (2014) applied a 5-HT1AR and 5-HT7R agonist (5-carboxamidotryptamine, 5-CT), no significant outward current was observed until P60, although P21 was trending toward significantly greater response than at P4. The 5-HT1AR response not only depends on expression of 5-HT1AR, but also on expression of the downstream G-protein signaling components. The authors used a non-hydrolyzable form of GTP that did not elicit an outward flow of current until P21 and P60. These results indicate that the genes for 5-HT1AR and the relevant G-protein signaling components are not fully activated until roughly the third week of life.

Rood et al. (2014) also evaluated 5-HT neuron dendritic morphology from P4 to P60. Initially at P4, dendrites in the dorsal raphe were shorter and more branched, then the dendrites grew longer until P21, then shortened dramatically by P60. Thus, DRN 5-HT neuron dendrites start out highly branched and seemingly undergo pruning late in maturation to become simpler in branching than P4 neurons with roughly equivalent length. Interestingly, MRN 5-HT neuron dendrites do not change dramatically from P4 to P60. In conclusion, the results found by Rood, et al., demonstrate remarkable alterations in electrophysiological properties and dendritic morphology of dorsal raphe neurons from the first week of life to adulthood representing a maturation process required for normal function in adulthood.

### 3.2. Maturation of morphology and connectivity

5-HT neurons establish expansive axonal connectivity patterns throughout the CNS. Just after 5-HT neurons are born, they begin sending axons into the brain and spinal cord and reach their target region between late embryonic through the third week of life (Lidov and Molliver, 1982). Surprisingly little is known about the mechanisms regulating 5-HT neuron axon outgrowth, axon guidance, terminal field recognition, and axon ramification. The growth-associated protein GAP-43 is highly expressed in 5-HT neurons and has a strong ascending gene expression trajectory (Wyler et al., 2016). In GAP-43-null mice, there are severe reductions of 5-HT fibers in terminal fields of the frontal cortex and hippocampus, with milder reductions in the piriform cortex and septum, while the ventrobasal thalamus is hyperinnverated (Donovan et al., 2002). The mechanism for how GAP-43 is involved in proper 5-HT neuron innervation patterns remains unknown, although it is possible that GAP-43-null 5-HT neurons are not able to sense NCAM, L1, or N-cadherin signals that could guide 5-HT axons (Meiri et al., 1998).

Another interesting study demonstrated that the Wnt planar cell polarity pathway is required for proper anterior-posterior (A-P) axon guidance of 5-HT neurons (Fenstermaker et al., 2010). The Wnt receptors Fzd3, Celsr3, and Vangl2 are expressed in 5-HT neurons and when deleted have various axon guidance defects. In each receptor mutant, ascending axons from B4-B9 5-HT neurons are partially misrouted laterally or posteriorly. Likewise, descending axons from B1-B3 5-HT neurons are misrouted anteriorly or have fasciculation defects. The Wnt5a ligand is expressed in opposing gradients with high expression at the isthmus decreasing to rhombomere 4, then increasing from r4 posteriorly. Thus, complex expression gradients of Wnt5a direct the correct A-P guidance of 5-HT axons early in 5-HT neuron maturation.

A recent study investigated the function of Ephrin/Eph signaling in 5-HT axon guidance and target region recognition (Teng et al., 2017). The authors showed strong expression of the EphA5 receptor in dorsal raphe 5-HT neurons. Then by knocking out EphrinA5 ligand globally, 5-HT neuron axons from the dorsal raphe aberrantly targeted the ventromedial hypothalamus and the external plexiform layer of the olfactory bulbs, which are normally targeted by median raphe neurons. Overexpression of the EphrinA3 ligand in the amygdala and piriform cortex resulted in reduced innervation of those regions, suggesting EphrinA signaling is sufficient to induce avoidance by 5-HT neurons. Thus, EphrinA/EphA signaling is a key repulsive cue for preventing dorsal raphe 5-HT neurons from innervating at least the ventromedial hypothalamus and external plexiform layer of the olfactory bulb that are normally only innervated by median raphe 5-HT neurons. However, much still remains unknown about the intrinsic molecular pathways controlling 5-HT axon guidance, terminal field recognition, axon branching, and 5-HT neurons special ability to regrow axons after injury (Jin et al., 2016). Furthermore, the transcriptional regulatory factors required to orchestrate these processes within the various raphe are unknown. Dissecting the roles of the transcription factors and their target pathways expressed during 5-HT neuron maturation will likely yield tremendous insight into the complex wiring pattern of 5-HT neurons in the CNS.

### 3.3. Gene expression trajectories

A key step in understanding the molecular and functional properties of a specific cell-type is developing a “parts list” comprised of expressed RNAs and proteins for that cell-type from birth of the cell throughout life. The first study to define 5-HT neuron cell-specific transcriptomes, used a combination of fluorescence-activated cell sorting (FACS) and microarray techniques to isolate 5-HT neurons and profile genome-wide gene expression patterns (Wylie et al., 2010). This study used a transgenic mouse line that expresses EYFP in 5-HT neurons under control of the Pet-1 enhancer region (ePet::EYFP), so the authors were able to microdissect the rostral and caudal portions of the hindbrain at E12.5, dissociate cells, then flow sort YFP+ neurons. Comparing expression data between the rostral and caudal samples revealed distinct markers for each group with Hmx2/3 marking rostral Pet-1+/5-HT neurons and a group of Hox genes marking caudal 5-HT neurons. In the rostral group, an additional subtype was defined by Engrailed gene expression resulting in Hmx^+^En^+^ and Hmx^+^En^−^ rostral subtypes.

Many studies have used transgenic mouse lines that express EYFP or Cre recombinase under the control of the Pet-1 enhancer region (ePet::EYFP, ePet::Cre, Scott and Deneris, 2005; Scott et al., 2005; Liu et al., 2010; Fox and Deneris, 2012). The ePet promoter/enhancer fragment in these constructs directs gene expression to the majority of 5-HT neurons in the various raphe nuclei (Scott et al., 2005; Zhao Z.-Q. et al., 2006). Some of these Pet-1+ cells do not present an overt 5-HT identity (Tph2+/5-HT+) when assayed with standard *in situ* hybridization or immunohistological protocols. The number of Pet-1+ cells without detectable 5-HT identity was reported to be about 27% in the MRN while in the B7 and B6 portions of the DRN this number was only 1% (Pelosi et al., 2014). These findings were interpreted as evidence that not all Pet-1+ cells are 5-HT neurons (Sos et al., 2016). However, recent single cell RNA seq studies have revealed that all of the single Pet-1+ cells sequenced in the DRN and MRN express *Tph2*, although some had a much lower number of *Tph2* reads that other Pet-1+ cells (Okaty et al., 2015). The Pet-1+ cells with low *Tph2* were all from the MRN and no Pet-1+ cell profiled from the DRN had a low number *Tph2* reads. This was corroborated with single molecule FISH. These findings indicate that the vast majority and perhaps even all Pet-1+ cell in the DRN and MRN are 5-HT neurons, but some do not express strong 5-HT (*Tph2*) identity. Thus, recent optogenetic studies of the DRN using ePet::Cre transgenic line were likely manipulating nearly exclusively 5-HT neurons (Liu et al., 2014).

The only study that profiled adult 5-HT neuron gene expression by microarray used the translating ribosome affinity purification method (TRAP, Dougherty et al., 2013). The TRAP method relies on expressing a GFP-tagged ribosomal protein subunit, L10a. The authors used a *Slc6a4* BAC to drive expression of the GFP-L10a construct in 5-HT neurons. Since L10a is a component of the ribosome, mRNAs that are being actively translated can be crosslinked to the ribosome and affinity-purified. The adult microarray expression profile overlapped significantly with the results from Wylie et al. (2010) despite the age difference and several sample preparation differences. Interestingly, when the investigators intersected their gene expression profile with data from the Autism Genetic Research Exchange (AGRE, Geschwind et al., 2001) they found a 5-HT neuron enriched gene, Celf6, with an inherited deleterious mutation in an affected individual. By generating a genetic knockout of Celf6 in mice, the authors found several autism-related phenotypic behaviors including resistance to change and decreased ultrasonic vocalizations. Intriguingly, the Celf6 knockout mice also had mildly decreased whole-brain levels of 5-HT, suggesting a disruption in 5-HT metabolism. Although Celf6 is predicted to be an RNA binding protein, it is not yet clear what role it plays in 5-HT neurons. Overall, the adult 5-HT neuron expression profile provides a useful dataset for testing gene function in adulthood.

**Figure 3:**
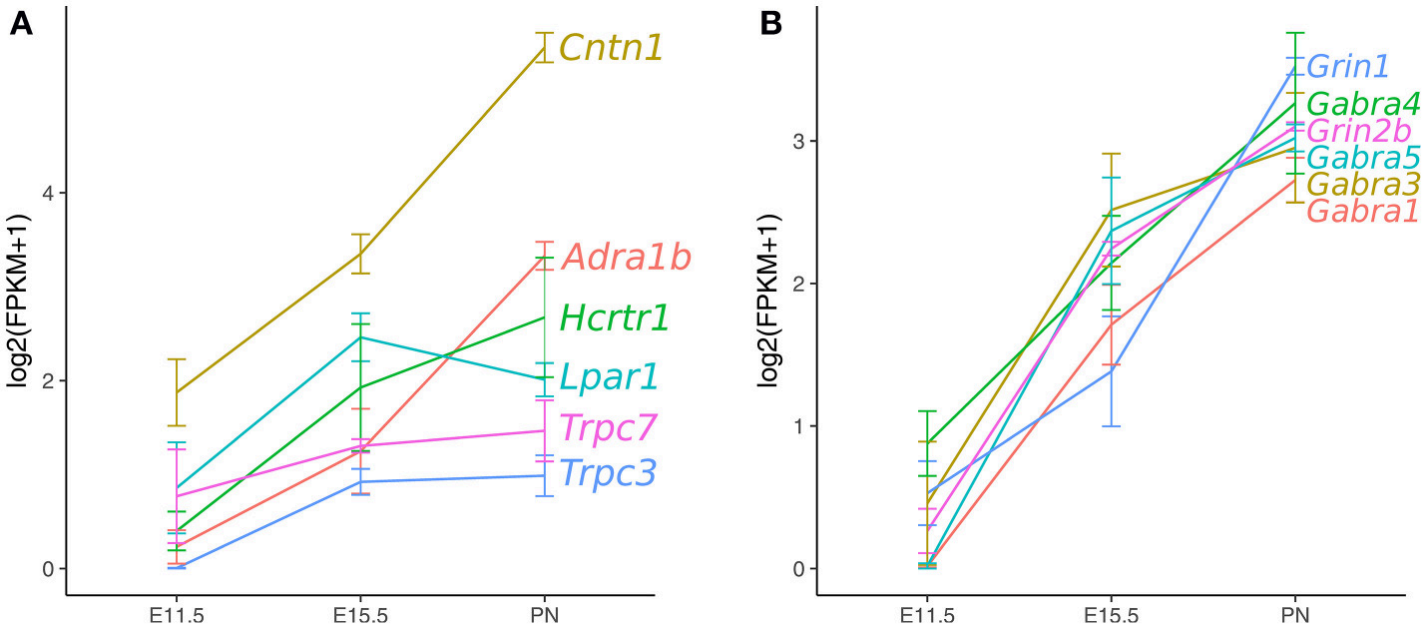
Ascending gene expression trajectories in maturing Pet-1+/5-HT neurons. RNA-seq analysis of Pet-1+/5-HT neurons at E11.5, E15.5, and postnatal time-points. **(A)** Axon guidance, GPCRs, and ion channels. **(B)** GABA-A and NMDA class glutamate receptor subunits. Expression values are log2(FPKM+1) and error bars are SEM (Wyler et al., 2016).

To define gene expression trajectories of maturing 5-HT neurons from early fetal to early postnatal stages, Wyler et al. (2016) isolated highly purified populations of Pet-1+ neurons from the rostral raphe nuclei (DRN and MRN combined) at E11.5, E15.5, and P1-P3 (PN) for RNA sequencing analysis. Hierarchical cluster analysis revealed genes that significantly changed in expression level through all three time points. The resulting clusters clearly delineated groups of genes with highly related ascending or descending temporal expression profiles. The clusters with descending trajectories contained genes with high expression at E11.5 and decreased through E15.5 to significantly lower levels by early postnatal stage. Gene ontology analysis showed that the genes in these clusters were enriched for transcriptional regulator, chromatin remodeler, and embryonic development genes that were expected for genes being highly expressed during the progenitor stage of 5-HT neurons. Concurrently, the gene clusters with ascending trajectories were off or lowly expressed at E11.5 and increased expression through the early postnatal stage. These clusters were enriched for genes involved in axonogenesis, ion channel activity, synaptic vesicles, and synaptic plasticity signifying the process of transitioning to a mature functional state of 5-HT neuron identity (Figure 3). While these results present a comprehensive transcriptional profile of 5-HT neuron maturation, they do not extend into the third week of life when Rood et al. (2014) showed 5-HT neurons are functionally mature. This can be resolved by considering the adult microarray profile (Dougherty et al., 2013), as well as, single-cell RNA-seq of 5-HT neurons at around 1 month of age (Okaty et al., 2015). The authors used manual sorting of neurons derived from r1, r2, r3, r5, and r6-r8 and performing RNA-seq on single cells or pools of cells, Okaty et al. (2015) provided a comprehensive transcriptional profile of multiple 5-HT neuron sub-types that are functionally mature. Indeed, many of the genes with ascending trajectories in Wyler et al. (2016), such as *Adra1b, Gria2, Lpar1*, and *Htr1a* are highly expressed in the data from Okaty et al. (2015). It is now clear that extensive temporal transcriptional changes underlie 5-HT neuron maturation, which raises the question of what regulatory strategies are employed to control these changes and promote the maturation process. Are terminal selector TFs involved in shutting off the early specification program and activating the later maturation program? Do other regulatory factors get activated early in maturation and drive higher gene expression in later ages?

## 4. Control of maturation by Pet-1

### 4.1. Functional properties

As discussed previously, Pet-1 functions as a terminal selector in 5-HT neurons by activating the core 5-HT neuron gene battery, autoregulating its own expression, and maintaining expression throughout the life of the cell (Deneris and Hobert, 2014). It was not known whether Pet-1 is involved in the functional maturation of 5-HT neurons. Previous work has shown that Pet-1-deficient neurons have increased spontaneous firing frequency and lack 5-HT1AR autoregulation (Liu et al., 2010). To determine if Pet-1 knockout neurons are functionally immature, Wyler et al. (2016) tested several passive and active membrane properties. Interestingly, Pet-1 knockout neurons had a depolarized resting membrane potential, increased membrane resistance, and an increased membrane time constant (tau, Table 2). These properties correspond with the previously reported immature 5-HT neuron phenotype at early postnatal ages (P4–P12, Rood et al., 2014). Action potential (AP) characteristics were also disrupted in Pet-1 knockout neurons. The AP amplitude was decreased with a hyperpolarized AP firing threshold. The afterhyperpolarization amplitude was also decreased. These passive and active membrane properties contribute to neuronal excitability and measuring current-induced excitability of *Pet-1*^−/−^ neurons demonstrated a permanent increase in excitability through adulthood. It is important to note that *Pet-1*^−/−^ neurons are maintained in the same numbers as in wildtype or control animals and do not appear to be directed to an alternative cell-fate. Therefore, the changes in functional properties are not due to recording from dying cells or adoption of functional properties of another neuron-type. These findings together reveal that Pet-1 function is not limited to the induction of 5-HT transmitter identity, but also for the acquisition of mature electrophysiological properties.

**Table 2:** Electrophysiological properties of *Pet-1*^−/−^ neurons (Wyler et al., 2016).

**Property**	**P21 +/+**	**P21 −/−**	**Adult +/+**	**Adult−/−**
Resting membrane potential	⇔	⇔	⇓	⇑
Membrane resistance	⇓	⇑	⇓	⇑
Membrane time constant (tau)	⇓	⇑	⇓	⇑
Firing rate	⇓	⇑	⇓	⇑
G-protein signaling	⇑	⇓	n/a	n/a
Action potential threshold	⇑	⇓	⇑	⇓
Afterhyperpolarization amplitude	⇓	⇑	⇓	⇑

*+/+ is wildtype and −/− is Pet-1^−/−^. Arrows indicate changes in properties and are relative to the alternate genotype at the same age. Left-right arrows indicate no difference. G-protein signaling was only assayed at postnatal day 21 (P21)*.

### 4.2. The Pet-1 regulon

The establishment of Pet-1 as a TF required for functional maturation raises the question of what gene categories are under control of Pet-1 during maturation. It is clear from the gene expression trajectories from E11.5 to the early postnatal period, 5-HT neurons undergo tremendous remodeling of their transcriptomes, while maintaining their core 5-HT transmitter identity (Figure 3). Wyler et al. (2016) tested whether Pet-1 controls maturation of gene expression trajectories and how any disruptions relate to the functional immaturity of Pet-1 deficient 5-HT neurons. Multiple broad categories of genes encoding GPCRs, ion channels, adhesion molecules, and transcription factors were found to be regulated by Pet-1, both positively and negatively. A significant proportion of these genes were also found to be directly bound by Pet-1 using a chromatin-immunoprecipitation sequencing (ChIP-seq) strategy. The disrupted expression of genes involved in many different 5-HT neuron relevant pathways emphasizes the broad regulatory role of Pet-1 in establishing the mature 5-HT neuron identity.

#### 4.2.1. GPCRs

G protein-coupled receptors (GPCR) represent a large category of proteins critical for various cellular functions such as neurotransmission. Many neurotransmitter receptors are GPCRs including most 5-HT receptors (5-HT1-2,4-7R). The 5-HT1AR receptor expressed in 5-HT neurons controls 5-HT neuron excitability through autoregulation. 5-HT1AR is coupled to the G_*i*_/*o* pathway and when 5-HT is released onto 5-HT neurons, 5-HT1AR receptors are activated, which activates G protein-coupled inwardly rectifying potassium channels (GIRK) in turn causing membrane hyperpolarization, which reduces or blocks action potential firing. 5-HT1AR activation also inhibits N- and P/Q-type voltage-gated Ca^2+^ channels reducing somatic Ca^2+^ currents. While it was known that Pet-1 transcriptionally regulates *Htr1a* (Liu et al., 2010), Wyler et al. (2016) showed using conditional targeting techniques that *Htr1a* is dependent on Pet-1 for expression through the late postnatal period, but loses complete dependency on Pet-1 in early adulthood (~2 months). It is not yet understood what molecular mechanisms underlie the loss of transcritional regulation of *Htr1a* by Pet-1. This result bears a striking similarity to the postnatal sensitivity period of 5-HT1AR expression (Donaldson et al., 2014; Rebello et al., 2014). Decreased expression of 5-HT1AR from P2 to P11 or P14 to P30 results in increased anxiety phenotypes later in adulthood. The dependency of 5-HT1AR expression on Pet-1 regulation during 5-HT neuron maturation, suggests a temporal window when perturbations of the transcriptional controls of *Htr1a* could lead to susceptibility for abnormal affective behaviors in adulthood.

**Figure 4:**
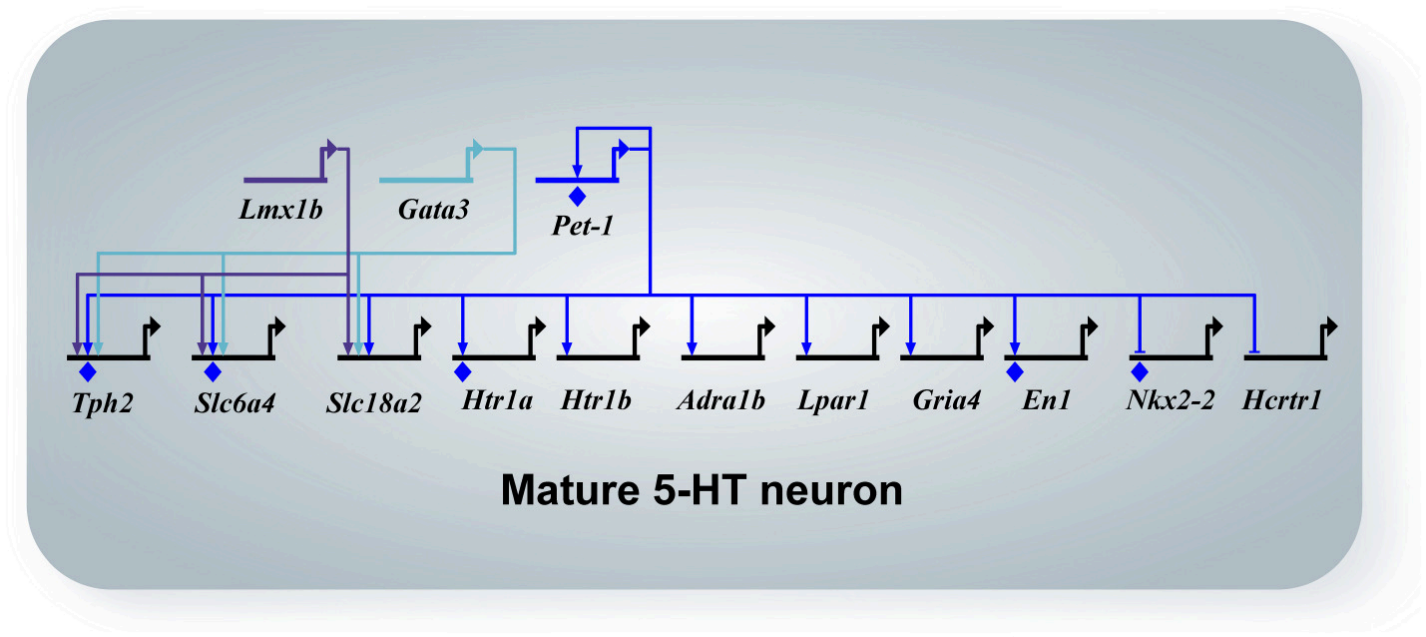
Network diagram of mature 5-HT neurons. All arrows indicate activation, horizontal marker indicates repression, and diamonds indicate direct binding of the transcription factor to the promoter of the target gene by ChIP experiments. Pet-1, Lmx1b, and Gata3 maintain expression of Tph2, Sert (*Slc6a4*), and Vmat2 (*Slc18a2*). Pet-1 activates the maturation genes *Htr1a, Htr1b, Adra1b, Lpar1, Gria4*, and represses *Nkx2-2* and *Hcrtr1*. Pet-1 directly regulates the maintenance factor *En1*.

Other interesting GPCRs identified in maturing 5-HT neurons include *Adra1b, Lpar1, Calcr, Mchr1, Tacr1*, and *Tacr3* (Wyler et al., 2016). These GPCRs have also been identified in adult Pet-1+/5-HT neurons by single-cell RNA-seq (Spaethling et al., 2014; Okaty et al., 2015). *Adra1b* encodes the α_1_*B* adrenergic receptor, which is not expressed at the time of 5-HT gene battery activation, but then progressively increases expression through fetal to the early postnatal period (Figure 3, Wyler et al., 2016). Noradrenergic neurons are known to stimulate tonic firing of 5-HT neurons through the α_1_ adrenergic receptor, and without this extrinsic control, 5-HT neurons no longer fire (Vandermaelen and Aghajanian, 1983). Pet-1 knockout neurons did not show an increase in excitability in response to phenylephrine, a selective α_1_ agonist. The lysophosphatidic acid receptor (LPA_1_) is a GPCR encoded by *Lpar1*, which increased expression significantly by E15.5 and maintained the same level through at least P3 (Figure 3). LPA signaling has been implicated in a diverse array of biological processes including neurite retraction, neuronal polarity, cell death/survival, and axon branching (Yung et al., 2015). *Lpar1* is highly dependent on Pet-1 for expression and loss of Pet-1 leads to a complete absence of increased firing rate of 5-HT neurons in response to increasing concentrations of an LPA_1_ agonist (Z)-N-[2-(phosphonooxy)ethyl]-9-octadecenamide (NAEPA) (Wyler et al., 2016). It is not currently known what the role for *Lpar1* is in 5-HT neurons, although it is interesting to note that in *Lpar1* knockout mice, there is a decrease in the 5-HIAA/5-HT ratio in the frontal cortex, hippocampus, hypothalamus, and nucleus accumbens indicating a disruption in 5-HT metabolism (Harrison et al., 2003). Thus, *Adra1b* and *Lpar1* provide clear examples of Pet-1's role in controlling 5-HT neuron maturation through control of specific GPCR effector genes (Figure 4).

#### 4.2.2. Ion channels/accessory proteins

5-HT neurons receive excitatory glutamatergic inputs in the raphe nuclei (Harsing, 2006) and have been shown to respond to AMPA/kainate and NMDA agonists (Gartside et al., 2007; Crawford et al., 2011; Maejima et al., 2013). The glutamate receptor subunit composition has not been well addressed in 5-HT neurons. In Wyler et al. (2016), it was found that the ionotropic AMPA receptor subunits *Gria2* and *Gria4* were the highest expressing genes by the early postnatal period. The other subunit genes, *Gria1* and *Gria3*, had very low expression levels in Pet-1+/5-HT neurons. The RNA-seq analysis and *in situ* hybridization of Pet-1 knockout neurons showed that expression of *Gria4* is lost in *Pet-1*^−/−^ neurons. In fact, electrophysiological analysis of *Pet-1*^−/−^ neurons revealed decreased excitatory postsynaptic current (EPSC) frequency variability, amplitude, decay time, and charge (Wyler et al., 2016). Thus, Pet-1 has a critical role in establishing mature ionotropic glutamatergic inputs of 5-HT neurons through control of at least *Gria4* (Figure 4).

The transient receptor potential (TRP) class of proteins constitute a diverse family of Ca^2+^ channels that are now known to be involved in many physiological processes (Nilius and Owsianik, 2011). While TRP channels are well-known for their function in sensory processes, they have other critical functions including ion homeostasis. TRP channels are expressed in a variety of cell types and have different activation, gating, and ligand-binding properties. In 5-HT neurons, three TRP channel genes have ascending expression trajectories into the postnatal maturation period and are also regulated by Pet-1 (Figure 3, Wyler et al., 2016). The three genes encode TRPC3, TRPC7, and TRPV2. TRPC channels are members of the canonical group, while TRPV channels are members of the vanilloid group. TRPC3 is known to functionally couple with the hypocretin/orexin receptor, *Hcrtr1*, which also has an ascending expression trajectory in 5-HT neurons (Figure 3, Peltonen et al., 2009). Pet-1 represses *Hcrtr1* and when Pet-1 is conditionally ablated postnatally, *Hcrtr1* expression strongly increases (Wyler et al., 2016). Hypocretin neurons in the hypothalamus drive an arousal/attention signal that triggers a bias in 5-HT neuron activity toward phasic over tonic firing (Gartside et al., 2000; Ishibashi et al., 2016). Therefore, Pet-1 may establish proper hypocretin responsiveness through moderating *Hcrtr1* and driving Trpc3 expression levels. TRPC7 is the newest member of the TRPC family and is activated by diacyl glycerol (DAG, Zhang and Trebak, 2014). Thus, TRPC7 may function as a non-selective Ca^2+^ channel downstream of the hypocretin receptor or another Gq-coupled GPCR in 5-HT neurons. TRPV2 was originally thought to be a noxious heat sensor, but in Trpv2 knockout mice, no thermosensation phenotype was observed (Park et al., 2011). Recently, TRPV2 has been shown to to be activated by nerve growth factor to promote neurite outgrowth of peripheral neurons during development (Cohen et al., 2015). With an ascending expression trajectory during maturation of 5-HT neurons, it is intriguing to suggest that TRPV2 may have a role in 5-HT neuron axonogenesis.

#### 4.2.3. Cell adhesion/axon guidance

The immunoglobulin superfamily of adhesion molecules represents a diverse and critical class of proteins involved in neuronal development, cell-cell interactions, and synaptic function. A subset of this family is the glycosyl-phosphatidyl inositol (GPI) anchored glycoproteins, Contactins (Gennarini et al., 2017). Contactins have C2-type Ig domains at their amino terminus and Fibronectin type III domains proximal to the plasma membrane. Contactins interact with other proteins heterophilically in *cis* and *trans* including L1CAMs and CNTNAPs (Contactin-associated proteins, also called Casprs). The Contactin-1 gene, *Cntn1*, is highly expressed in 5-HT neurons with a strong ascending gene expression trajectory into the postnatal period (Figure 3, Wyler et al., 2016). *Cntn1* expression is also greatly decreased in *Pet-1* knockout 5-HT neurons. *Cntn1* mutant mice have a severe phenotype with late postnatal lethality and ataxia (Berglund et al., 1999). The cerebellum has defects in granule cell axon guidance as well as granule and Golgi cell dendritic projections. Contactin-1 has been shown to be characteristically expressed in maturing neurons and is localized to axonal surfaces in an activity-dependent manner (Gennarini et al., 1989a,b; Pierre et al., 2002). It is not known what function Contactin-1 has in 5-HT neurons, yet given the broad connectivity pattern and vast array of cellular interactions, it would be intriguing to investigate the role of Contactin-1 in maturing 5-HT neurons. The CNTNAP gene *Cntnap3* also has an ascending gene expression trajectory and is positively regulated by Pet-1 (Wyler et al., 2016). Not as much is known about Cntnap3 (Caspr-3), although *Cntnap3* mutant mice have a defect in motor learning and it was suggested to be involved in NMDA receptor localization in the striatum (Hirata et al., 2016). Cntnap3 could potentially interact with Contactin-1 in *cis* or other adhesion molecules in *trans* to regulate certain aspects of 5-HT neuron maturation and connectivity. Interestingly, both Contactins and CNTNAPs have been associated with Autism spectrum disorder, in particular CNTNAP2, although another report has associated a mutation in CNTNAP3 with Asperger's syndrome (Strauss et al., 2006; Alarcón et al., 2008; Arking et al., 2008; Bakkaloglu et al., 2008; Peñagarikano et al., 2011; Vaags et al., 2012). Recently, CNTNAP2 has been shown to bind to CNTN1 suggesting CNTN1 is likely the endogenous ligand for CNTNAP2 (Rubio-Marrero et al., 2016). Additional investigation will be required to determine the roles for these adhesion molecules in the 5-HT system and if disruption in their function during 5-HT neuron maturation could play a role in Autism-related disorders in humans.

#### 4.2.4. Peptides

In addition to 5-HT as the defining neurotransmitter of 5-HT neurons, subsets of 5-HT neurons also utilize neuropeptides for signaling. The caudal raphe 5-HT neurons (B1-B3) have been shown to express substance P, thyrotropin-releasing hormone (TRH), enkephalin (ENK), and calcitonin gene-related peptide (CGRP) (Hokfelt et al., 2000). In contrast to other species, in the rostral raphe (B4-B9) of the mouse, only rare overlap has been shown between neuropeptide expression and 5-HT (Fu et al., 2010). However, it has now been shown that rostral 5-HT neurons express several neuropeptide transcripts using RNA-seq including *Npy, Nts, Pdyn, Sst, Trh*, and *Penk* (Okaty et al., 2015; Wyler et al., 2016). Interestingly, *Npy, Nts, Pdyn, Sst*, and *Penk* have ascending gene expression trajectories in rostral 5-HT neurons and are regulated by Pet-1, both positively (Npy, Nts, Pdyn, and Sst) and negatively (Penk) (Wyler et al., 2016). The reason for the discrepancy between the immunohistochemical staining for these peptides in Fu et al. (2010) and the RNA-seq data in Okaty et al. (2015) and Wyler et al. (2016) is not clear, although immunodetection for peptides may lack the sensitivity for detection that RNA-seq provides. Additional studies including functional approaches to test for roles of neuropeptide signaling in rostral 5-HT neurons will be required to resolve this issue.

#### 4.2.5. Network of transcription factors in postmitotic neurons

As discussed earlier in this review, numerous transcription factors are involved in the specification and terminal differentiation of 5-HT neurons. As a terminal selector in 5-HT neurons, Pet-1 likely functions with co-activators and repressors to establish 5-HT neuron specific features as well as general neuronal features (Deneris and Hobert, 2014). This transcriptional regulatory strategy is also able to generate heterogeneity within a cell-type, which is likely required to obtain the morphological, molecular, and functional diversity observed in 5-HT neurons. Many transcription factors have now been identified using ChIP-seq that are likely directly regulated by Pet-1 (Wyler et al., 2016). These include known 5-HT neuron TFs, such as, En1 and Nr3c1 (glucocorticoid receptor, GR) and many other TFs with unknown function in 5-HT neurons. En1 has been shown to be required for proper migration of DRN 5-HT neurons toward the midline and maintenance of 5-HT neurons postnatally (Fox and Deneris, 2012). The glucocorticoid receptor is well-known for its role in sensing stress levels through binding its ligand cortisol (corticosterone in rodents) and mediating glucocorticoid feedback regulation of the hypothalamic-pituitary-adrenal axis. Transcriptional regulation by GR can vary greatly depending on binding partners and DNA regulatory elements. GR has been deleted from DRN cells (including 5-HT neurons) in a non-cell-specific manner which led to diminished dysphoria-like behavior (Vincent and Jacobson, 2014). To determine if GR has a cell-autonomous role in 5-HT neurons, it will be necessary to delete GR in a cell-specific manner using available tools (Scott et al., 2005; Liu et al., 2010). Nonetheless, GR may interact with Pet-1 or other TFs to control stress-related functions or drive maturation in 5-HT neurons. A known transcriptional co-activator, Cited1, was strongly expressed throughout 5-HT neuron maturation and was greatly decreased in Pet-1 mutant neurons. Another interesting target of Pet-1 is *Nkx2-2*. Nkx2.2 plays an important role in 5-HT neuron progenitors to regulate the timing of the switch between production of visceral motor neurons and 5-HT neuron precursors and has been shown to induce Gata2 and Gata3 expression in chick rhombomere 1 (reviewed in Deneris and Wyler, 2012). In rhombomere 4 that does not produce 5-HT neurons, Nkx2.2 activates Phox2b to repress Foxa2 which represses the 5-HT neuron fate. *Nkx2-2* shows a decline in expression in 5-HT neurons from E11.5 through the early postnatal period, but is derepressed in Pet-1 knockout 5-HT neurons (Figure 4, Wyler et al., 2016). This suggests Pet-1 represses an early cell-fate TF allowing 5-HT neurons to proceed into maturation. With the discovery of many TFs expressed in maturing 5-HT neurons controlled by Pet-1, it is now possible to test other TFs for a function in regulating maturation of 5-HT neuron morphology and function.

### 4.3. The role of transcriptional activation and repression in 5-HT neuron identity

Cell-types within the nervous system are categorized based on a variety of characteristics including location, morphology, neurotransmitter usage, activity, and gene expression. Investigation of how these specific cell-types arise is often focused on identifying a transcription factor that is required to activate the genes required for that cell-type. Each cell-type, however, may be broad in definition with many distinct sub-types. These sub-types may have different morphologies, alternate release or co-release of other transmitters, and functional characteristics. 5-HT neurons are such a class of neurons with a variety of sub-types (Wylie et al., 2010; Spaethling et al., 2014; Okaty et al., 2015; Fernandez et al., 2016). 5-HT neurons can be divided by rostral vs. caudal projections, brain region specific innervation patterns, afferent inputs, vesicular glutamate transporter expression (vGlut3+), neuropeptide usage, and electrophysiological properties. The regulatory network strategies for creating such diversity are not well elucidated. A recent study by Kerk et al. (2017), investigated the regulatory strategy controlling cholinergic neuron diversity in *C. elegans*. The cholinergic motor neuron terminal selector UNC-3 regulates ACh pathway genes, ion channels, signaling proteins, and neurotransmitter receptors in these neurons, yet different sub-types are defined by anatomical and functional traits. The terminal selector TF, UNC-3, is expressed in all of these neurons, therefore it is not able to selectively activate these different features alone. The expression of co-activators or repressors are two possible strategies for generating the various sub-types. Kerk et al. (2017) use genetic screens to test whether either or both strategies are utilized in *C. elegans* cholinergic motor neurons. Their study revealed a surprisingly consistent use of repressors to inhibit specific genes from being activated by UNC-3 in the different sub-types. Thus, UNC-3 has the role of broadly activating cholinergic neuron genes and then other repressor proteins override the UNC-3 program to diversify cholinergic neuron sub-types. In contrast, Pet-1 functions as a terminal selector for mouse 5-HT neurons and has shown to act as both an activator and repressor (Fyodorov et al., 1998; Maurer et al., 2003; Wyler et al., 2016). Pet-1 represses *Nkx2-2, Hcrtr1*, and *Penk* as mentioned above. *Penk* encodes the enkephalin peptide and is highly expressed in caudal 5-HT neurons (Okaty et al., 2015). This suggests Pet-1 is repressing a caudal 5-HT neuron gene to separate the rostral and caudal sub-types. Pet-1 expression is maintained in caudal 5-HT neurons, so it is unclear how *Penk* expression is increased in caudal neurons relative to rostral neurons. Other transcription factors that are expressed and/or regulated by Pet-1 in 5-HT neurons likely contribute to 5-HT neuron heterogeneity including *Lmx1b, Gata2, Gata3, En1*, and others described above (Ding et al., 2003; Zhao Z.-Q. et al., 2006; Zhao et al., 2007; Song et al., 2011; Yan et al., 2013; Haugas et al., 2016).

## 5. Conclusions and future directions

5-HT neurons lacking Pet-1 through global or conditional deletion have been molecularly and physiologically profiled. In general, when an important cell-fate specification TF is deleted, a frequent observation is that the cells undergo apoptosis or adopt an alternate cell fate. With the exception of some neurons in B6, nearly all *Pet-1*^−/−^ neurons are maintained and it is not clear whether B6 5-HT neurons die in *Pet-1*^−/−^ animals or are mislocated to more anterior parts of the DRN. Also, there is no readily apparent adoption of an alternative cell fate, such as a motor neuron identity. Since Pet-1 plays a major role in maturation of 5-HT neurons, are *Pet-1*^−/−^ neurons simply generic immature neurons lacking a dominant neurotransmitter identity? The majority of *Pet-1*^−/−^ neurons no longer produce 5-HT, although some maintain enough expression of the 5-HT gene battery to continue synthesis (Kiyasova et al., 2011), likely due to continued expression of Lmx1b. Some subsets of 5-HT neurons primarily in the medial DRN and MRN also express the vesicular glutamate transporter type 3 (vGlut3) enabling the packaging of glutamate into vesicles for release (Rood et al., 2014; Haugas et al., 2016). In *Pet-1*^−/−^ neurons, vGlut3 expression is decreased likely resulting in decreased ability to package and release glutamate in that subset neurons. Interestingly, many genes expressed at higher levels in caudal 5-HT neurons are derepressed in rostral *Pet-1*^−/−^ neurons (Wylie et al., 2010; Wyler et al., 2016). These include *Penk, Tac1, Calb2, Gabra5, Foxp2, Ebf1/2/3, Meis2*, and *Onecut2*. The *Tac1* gene encodes the neuropeptides substance P, neurokinin A, and neuropeptide K. *Gabra5* encodes a subunit of the GABA-A receptor. *Foxp2, Ebf1/2/3, Meis2*, and *Onecut2* are transcription factors that may activate neuropeptide gene expression in caudal 5-HT neurons or other caudal 5-HT neuron genes.

Several electrophysiological properties of dorsal raphe *Pet-1*^−/−^ neurons are also altered as discussed earlier in this review. The active and passive membrane properties of dorsal raphe *Pet-1*^−/−^ neurons are characteristic of immature 5-HT neurons (Rood et al., 2014; Wyler et al., 2016). The loss of important ion channels, receptors, and other molecules discussed in previous sections above likely result in this hyper-excitable, immature neuron phenotype. In total, *Pet-1*^−/−^ neurons do not synthesize, package, release, or sense 5-HT. They appear stuck in an immature hyper-excitable state and potentially release neuropeptides ectopically in regions where axons are present due to increased expression of neuropeptide genes. In the future it would be interesting to address whether the rostral *Pet-1*^−/−^ neurons that have increased neuropeptide expression also have altered axon projections and send their axons into the spinal cord, further suggesting the adoption of the caudal 5-HT neuron cell fate.

5-HT neurons of the dorsal and median raphe nuclei have a number of interesting differences in their projection patterns, functional properties, and gene expression patterns. The MRN 5-HT neurons predominately project to midline structures, hippocampus, and other regions, whereas DRN 5-HT neurons project to the cortex, ventral midbrain, lateral hypothalamus, midline thalamus, amygdala, and other regions (Vertes and Linley, 2007; Muzerelle et al., 2014). Also, DRN and MRN 5-HT neurons largely do not overlap in their target regions. During maturation from early postnatal to adulthood, DRN 5-HT neurons show many electrophysiological changes including membrane potential, resistance, AP threshold, excitability, and 5-HT1AR response, whereas MRN 5-HT neurons have a decrease in membrane resistance, but not membrane potential or excitability (Rood et al., 2014). DRN 5-HT neurons are also strongly sensitive to 5-HT1AR autoregulation in adulthood, yet MRN 5-HT neurons have only a mild 5-HT1AR response. There is less known about differences in gene expression between DRN and MRN 5-HT neurons, although one notable difference is a larger proportion of *Slc17a8*+ neurons in the MRN vs. DRN (Okaty et al., 2015). It would be intriguing to see if there is a less dramatic change in the transcriptome of MRN 5-HT neurons during maturation compared to DRN 5-HT neurons, since MRN 5-HT neurons have fewer functional changes during maturation.

Following the terminal specification of 5-HT neuron cell fate, the process of maturation progresses nearly into adulthood. From early acquisition of the molecular machinery needed to synthesize, package, release, and sense 5-HT, to the integration into functional CNS circuitry, 5-HT neurons undergo many dynamic alterations during maturation. The gene regulatory networks orchestrating these processes are beginning to be elucidated with the terminal selector Pet-1 playing a critical role in establishing proper synaptic inputs and functional properties through activation and repression of target genes in a temporal manner. To further unravel the complexity of Pet-1+/5-HT neuron maturation, it will be necessary to address the roles of the other transcriptional regulators discovered in mature 5-HT neurons (Wylie et al., 2010; Dougherty et al., 2013; Okaty et al., 2015; Wyler et al., 2016). As the role of these maturation factors becomes more clear, the knowledge gained will also help determine the state of maturity of induced 5-HT neurons. Concomitantly, mature induced 5-HT neurons will likely better approximate endogenously generated 5-HT neurons and have greater utility for candidate gene experiments identified from genome-wide association studies of anxiety and affective disorders.

Studies of neuronal development have largely focused on mechanisms governing early patterning and cell-fate specification. In contrast, only a modest amount of research has investigated the cellular and molecular processes occurring between the acquisition of neuronal identity features and the attainment of mature morphological and functional characteristics. Several studies have focused on the maturation of GABA neurons in the cortex and hippocampus, as well as, adult-born granule neurons in the dentate gyrus (Liu et al., 1996; Ye et al., 2000; Ambrogini et al., 2004; Zhao C. et al., 2006; Butt et al., 2007; Villette et al., 2016). These studies and others have noted many dynamic changes in transcriptome profiles, cell morphology, synaptic properties, and intrinsic physiology during maturation as is now known for 5-HT neurons. In the future, it will be necessary to utilize a broad range of experimental approaches to further our understanding of the processes that underlie neuronal maturation, and if perturbed during development, what is the lasting effect on behavior or mental health.

## Author contributions

WS conceived the content and wrote the manuscript. ED conceived the content and advised the writing of the manuscript. Both authors read and approved the final manuscript.

### Conflict of interest statement

The authors declare that the research was conducted in the absence of any commercial or financial relationships that could be construed as a potential conflict of interest.
